# Miniaturized Protein Microarray with Internal Calibration as Point-of-Care Device for Diagnosis of Neonatal Sepsis

**DOI:** 10.3390/s120201494

**Published:** 2012-02-03

**Authors:** Patricia Buchegger, Ursula Sauer, Hedvig Toth-Székély, Claudia Preininger

**Affiliations:** Health & Environment Department, AIT Austrian Institute of Technology, Bioresources, Konrad Lorenz Straße 24, 3430 Tulln, Austria

**Keywords:** protein microarray, streptavidin coated magnetic particles, miniaturization, point-of-care testing, neonatal sepsis, internal calibration

## Abstract

Neonatal sepsis is still a leading cause of death among newborns. Therefore a protein-microarray for point-of-care testing that simultaneously quantifies the sepsis associated serum proteins IL-6, IL-8, IL-10, TNF alpha, S-100, PCT, E-Selectin, CRP and Neopterin has been developed. The chip works with only a 4 μL patient serum sample and hence minimizes excessive blood withdrawal from newborns. The 4 μL patient samples are diluted with 36 μL assay buffer and distributed to four slides for repetitive measurements. Streptavidin coated magnetic particles that act as distinct stirring detection components are added, not only to stir the sample, but also to detect antibody antigen binding events. We demonstrate that the test is complete within 2.5 h using a single step assay. S-100 conjugated to BSA is spotted in increasing concentrations to create an internal calibration. The presented low volume protein-chip fulfills the requirements of point-of-care testing for accurate and repeatable (CV < 14%) quantification of serum proteins for the diagnosis of neonatal sepsis.

## 1. Introduction

Sepsis is a life-threatening systemic body infection manifested by symptoms ranging from elevated heartbeat, breathing difficulties and fever to severe necrosis of the internal organs. It is caused by microbial pathogens including bacteria, virus and fungi. The survival rates increase enormously with an early stage diagnosis which allows timely administration of antibiotics. Neonatal sepsis is still a leading cause of death among newborns since symptoms are often wrongly interpreted due to their unspecific and late occurrence [1]. However, early-stage diagnosis in neonates is even more challenging and demands for a reliable and rapid point-of-care device that works with a low sample volume to keep invasive procedures on neonates to a minimum. Key requirements for point-of-care devices relate to short assay time, quality assurance through simple and stable calibration, convenient operation and cost effectiveness [2]. The standard diagnosis procedure for sepsis is based on the isolation of microorganisms from patient blood or urine samples and subsequent microbiological cultivation and identification. Disadvantages of the standard procedures are the low sensitivity, the processing time which may exceed 24 hours and the need for large sample volumes (>1 mL) [3–5].

Sepsis is a condition caused by pathogenic microorganisms but induced by the immune response through excessive release of growth factors, cytokines and inflammation- and coagulation factors into the systemic circulation. Those factors can be exploited as specific biomarkers enabling early and reliable diagnosis. The cytokines interleukin 6 (IL-6), interleukin 8 (IL-8), interleukin 10 (IL-10) and tumor necrosis factor alpha (TNF alpha) and the serum proteins C-reactive protein (CRP), procalcitonin (PCT), S-100 and E-Selectin have been identified as a specific and accurate biomarker panel for the early diagnosis of sepsis. The clinically relevant ranges as well as the cut-off values of the respective serum proteins that allow discrimination between healthy and septicaemic patients are outlined in Table 1. IL-6, IL-8, IL-10 and TNF alpha are proinflammatory cytokines secreted by cells of the immune system that mediate host defense [6,7]. E-Selectin is expressed by endothelial cells and is a main mediator in the migration of immune cells to the centre of infection [8]. CRP and PCT are acute-phase reactants expressed predominately as part of the immediate immune response to infections [9]. S-100 serum levels are significantly elevated in neuronal inflammations [10]. Neopterin is expressed by macrophages in response to infections [11].

Herein we aim at the further development of a sepsis chip for point-of-care testing of neonatal sepsis. This is achieved by: (a) the reduction of required patient sample volume, (b) the reduction of assay steps and incubation times and (c) the integration of an internal calibration. The chip for biomarker detection of sepsis comprises multi-analyte on-chip immunoassays which include both sandwich (cytokines, PCT, S-100, E-Selectin) and binding inhibition formats (CRP, neopterin). The combination of both assay formats allows analysis of the selected biomarkers on one chip despite the large heterogeneity of the analytes regarding cut-off concentration [14]. In our previous work we showed that LODs of 15 pg/mL (IL-6), 36 pg/mL (IL-8), 65 pg/mL (IL-10), 40 pg/mL (TNF alpha), 0.078 ng/mL (PCT), 0.46 ng/mL (neopterin) and 3 μg/mL (CRP) are achieved in this set-up [14].

In order to prevent excessive blood withdrawal from newborns, the biochip was further miniaturized to operate with a five-fold reduced sample volume. The assay performance of the miniaturized chip is then compared with the classical format and evaluated using assay parameters like analyte concentration halfway between the zero point and the upper asymptote (IC_50_), limit of detection (LOD), limit of quantification (LOQ), and inter- and intra-array variations.

In general, assay miniaturization provokes loss in assay sensitivity due to e.g., diffusion limitations. While a sample volume of 50 μL, as used for the classical chip, is mixed properly during incubation on the rotational shaker, a sample volume of 10 μL, as used for the miniaturized chip, is too small to be moderately mixed under the same incubation conditions. Different theoretical considerations have been made concerning the impact of diffusion on the antibody antigen kinetics for immunoassays on solid surfaces [15–17]. For example, Kusnezow *et al.* [15] developed a mathematical model to simulate the impact of diffusion on the binding kinetics. This model was examined empirically comparing the development of signal intensities over time for anti-IFN-γ spots of 45 μm to 272 μm radius under stirring and non-stirring conditions. Clearly, stirring significantly accelerates the immunoreaction (up to hundreds of times), while the velocity increases with decreasing size of the spot. Hartmann *et al*. reported three-fold enhanced signals using surface acoustic waves (SAW) coupled through the glass slide into the reaction solution. To account for the antibody spot kinetics, thus the migration of the analytes in solution towards the spot and across the surface we included bi-functional streptavidin coated magnetic particles (Strep.MPs), which act as both micro-mixer and detection reagent for the biotinylated antibodies. Strep.MPs in the sizes of 130 nm, 500 nm and 1 μm were tested. In addition, the assay procedure was further optimized by reducing assay steps and incubation times. Furthermore the calibration was integrated on the chip (internal calibration) presenting a point-of-care device suitable for use in neonates.

## 2. Experimental Section

### 2.1. Materials and Reagents

Chip platform used was the proprietary ARChip Epoxy [14], Anti IL-6 (MQ2-13A5), recombinant IL-6 protein, biotinylated anti IL-6 (MQ2-39C3) as well as anti IL-10 (JES3-9D7), recombinant IL-10, biotinylated anti IL-10 (JES3-12G8), anti TNF alpha (MAb1), recombinant TNF alpha and biotinylated TNF alpha (MAbF6C5) were purchased from eBioscience (San Diego, CA, USA). Anti S-100 (MAb8B10), S-100 protein, biotinylated anti S-100 (MAb6G1), anti PCT (MAb16B5), biotinylated anti PCT (MAb42) and CRP-free serum were obtained from Hytest (Turku, Finland). Anti E-Selectin, human E-Selectin, biotinylated anti E-Selectin were purchased from R&D Systems (Minneapolis, MN, USA). Anti CRP (C5) and biotinylated anti CRP (C7) were from Exbio (Vestec, Czech Republic). Human procalcitonin was obtained from ProSpec-Tany TechnoGene Ltd. (Rehovot, Isreal). Neopterin conjugated with bovine serum albumin (BSA) and antibodies mAb 3E2 were kindly provided by Milan Franek, Veterinary Research Institute, Brno, Czech Republic and labelled with Dy647 by Exbio. Dy647 Streptavidin was from Dyomics (Jena, Germany) and Cy3-Streptavidin was from GE Healthcare (Chalfont St Giles, UK). Streptavidin coated magnetic particles with a 1 μm diameter were purchased from Roche (Basel, Switzerland), Streptavidin coated magnetic particles with a 500 nm and a 130 nm diameter were from Spherotech Inc. (Lake Forest, IL, USA) and magnetic particles with a 500 nm diameter were from micromod (Rostock-Warnemuende, Germany). Biotinylated anti Cy3/Cy5 (Cy-96), Tween 20, CHAPS, glycidyloxypropyltrimethoxysilane (GOPS), polyethylenglycol, MW 6000 (PEG 6000), ethanolamine and sodium deoxycholate were purchased from Sigma (St. Louis, MO, USA). 1-Ethyl-3-(3-dimethylaminopropyl)carbodiimide (EDC) was from Fluka Biochemicals (St. Louis, MO, USA). Phosphate buffered saline (PBS, pH 7.2) was purchased from Gibco (Invitrogen, Grand Island, NY, USA) and MES buffered saline packs were from Thermo Fisher Scientific (Portsmouth, NH, USA).

### 2.2. Dy647 Streptavidin Conjugation to Magnetic Nanoparticles

All washing steps were performed via centrifugation at 16,200 g-force number for 6 min (Eppendorf Centrifuge 5417C). Five hundred μL of a 1 mg/mL 500 nm magnetic particles suspension are coated with functionalized silica by adding 200 μL GOPS and sonicating for 90 min, then an additional 10 μL of GOPS was added and the particles were sonicated (Branson Ultrasonics B.V. 2510, Soest, The Netherlands) for another 90 min. The particles were washed twice with 1× PBS and resuspended in 1× PBS. Particles were washed 3× with 0.5 mM MES buffer (pH 5.0) by centrifugation and resuspended in 250 μL MES buffer. 50 μL Dy647-streptavidin (1 mg/mL Stock) and 50 μL PEG 6000 were added and incubated for 2 h on a rotational shaker at maximum speed (Stovall, USA). The reaction was vortexed every 30 min. The particles were washed twice with PBS and resuspended in 30 mM ethanolamine containing 1% BSA for 30 min to quench the reaction. The particles were washed 3× and resuspended in 500 μL 1× PBS containing 0.05% Tween 20 and 0.1% BSA (pH 7.4) and stored at 4 °C until use. The particle concentration was estimated to be 200 μg/mL. The protocol was adapted from [18].

### 2.3. Chip Fabrication

Capture antibodies were diluted in printing buffer “na” (1× PBS (pH 7.2)/0.01% Na-deoxycholate) to concentrations of 0.4 μg/mL for IL-6, IL-8, IL-10 and 0.5 μg/mL for TNF alpha, S-100, procalcitonin and E-Selectin. The proteins CRP and neopterin conjugated to BSA were diluted in printing buffer “cb” (1× PBS (pH 7.2)/0.005% CHAPS, 0.01% BSA) to concentrations of 0.5 mg/mL each. The appropriate spotting conditions (composition of print buffer, humidity during spotting and antibody concentration) were established in previous research [19]. The proteins were arrayed on proprietary ARChip Epoxy slides using the Omnigrid contact spotter from GeneMachines (pin SMP3). The spot-to-spot distance was 350 μm. Each of the probes was spotted in triplicates in 12 identical arrays at a relative humidity of 50%. To allow full immobilization of the probes the slides were kept at 4 °C for at least three days.

### 2.4. Internal Calibration Curve

S-100 protein was conjugated to BSA to improve immobilization on ARChip Epoxy. The conjugate was prepared by incubating 100 μL S-100 (0.5 mg/mL Stock) with 1 mg EDC for 10 min on an orbital shaker. An additional 10 μL BSA (5 mg/mL) were added and the reaction was allowed to proceed for 4 h. Zero to 218.7 ng/mL S-100-BSA-conjugate in buffer “cb” was then spotted onto the chip.

### 2.5. Chip Processing

Surface blocking was performed for 30 min in 1× PBS (pH 7.2)/0.1% Tween 20 in order to remove any unbound probes and to deactivate reactive surface groups. Slides were washed twice in 1× PBS (pH 7.2) and dried using compressed air. Assay buffer used consists of CRP free human serum and 0.1 M Tris (pH 7.4), 10 mM CaCl_2_, 100 mM NaCl, 0.1% Tween in a ratio of 1:10. This corresponds to 20 μL patient serum sample diluted with 180 μL Tris buffer for the classical chip and 4 μL patient serum sample diluted with 36 μL Tris buffer for the miniaturized chip.

#### 2.5.1. Classical Chip

The protocol of the “classical sepsis chip” has been introduced before [14]. Briefly, for one experiment a 4-slide set was mounted into the FastFrame (Whatman) to generate 4 × 12 array fields (7 mm × 7 mm each). This set accomodates 12 replicate measurements (four arrays with three replicate spots each) of nine point calibration standards and three “patient samples”, which are distributed on the slides taking into account the variation of production parameters. Each array was incubated with either 50 μL diluted sample or a calibration standard for 2.5 h.

Calibration standards were prepared by serial dilution of mixes of analytes in CRP-free serum and diluted with assay buffer 1:10. For the so called “external calibration” nine spiked standards were applied. After washing three times with 1× PBS (pH 7.2)/0.1% Tween-20, slides were incubated for 45 min with 50 μL biotinylated antibody mixes with end concentrations of 1 μg/mL each. After washing three times, slides were incubated with 4 μg/mL Dy647 streptavidin for 45 min. All incubation steps were carried out on the orbital shaker (Stovall) at maximum speed at room temperature. Slides were washed and dried using compressed air and stored in the dark until scanning.

#### 2.5.2. Miniaturized Chip

While the classical chip was processed in the FastFrame that provides wells that are 7 mm in length and width the low volume chip was arranged in silicon miniwells (GraceBio Laboratories, city, country) with incubation chambers of 4.5 mm in diameter. Those wells were small enough for a sample of 10 μL to completely cover the wells harboring the arrays. 10 μL patient serum sample or standard dilution series diluted 1:10 with assay buffer was incubated for 2.5 h in a humid chamber on the magnetic stirrer (Variom, Poly 15) at 450 rpm to allow sample mixing. The standard dilution series was spiked with appropriate amounts of antigen, biotinylated antibodies (1 μg/mL) and strep.MPs. (4 μg/mL). Detection occured via labeling with a biotinylated antibody against Cy3/Cy5 and Cy3 labeled streptavidin (1 μg/mL each). Slides were washed and dried using compressed air and stored in the dark until scanning.

### 2.6. Fluorescence Scanning

Fluorescence signals (λ_ex_: 635 nm, λ_em_: 670 nm) were recorded using the Genepix 4000B non-confocal scanner (Axon Instruments, Sunnyvale, CA, USA). Data were analyzed with the Genepix 6.0 software. The photomultiplier tube (PMT) voltage was held constant throughout the scans for each analyte to allow data comparison.

### 2.7. Data Analysis

The mean signal values were calculated from 12 replicates and were background corrected. Data that were out of the mean signal values ± the standard deviation (SD) were excluded. Standard curves were set up with GraphPad Prism 5.0 using the five parameter logistic fit.

The IC_50_ value (also reported as ED_50_) is defined as the concentration halfway between the zero point and the upper asymptote when the value of the asymmetric factor is 1. The limit of detection (LOD) is defined as lowest concentration that can be detected and was calculated by the mean zero concentration + three standard deviations (SD) of the blank [20]. The limit of quantification (LOQ) was defined as the lowest analyte concentration that could be reliably and quantitatively determined and was calculated by the mean zero concentration + 10 standard deviations of the blank [20]. The inter-array variability refers to the variation between the arrays (CV_inter_% = SD/mean × 100) and the intra-array variability to the variation within one array (CV_intra_% = SD of one triplicate/mean of one triplicate × 100). Recoveries were calculated to identify the accuracy of the assay displaying the % relation between the calculated concentrations and the expected concentrations.

## 3. Results and Discussion

### 3.1. Comparison of Chip Formats: Classical versus Miniaturized

**Figure 1.**
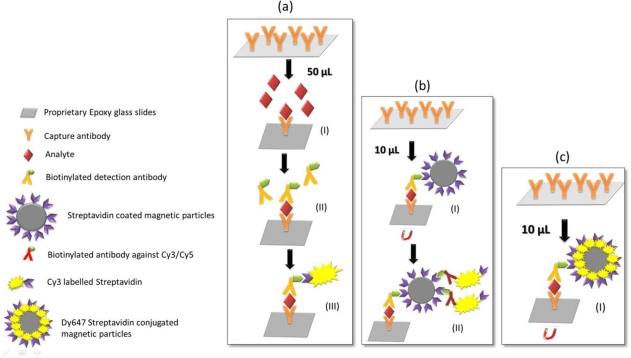
On-chip sandwich immunoassay steps for (**a**) the classical chip (**b**) the miniaturized chip and (**c**) the optimized miniaturized chip. Capture antibodies are immobilized equally providing same array format for the classical chip and the miniaturized chip. a(I) incubation of analytes (2.5 h), a(II) incubation of biotinylated detection antibodies (45 min), a(III) detection of bound antigens/antibodies via Dy647 Streptavidin incubation (45 min); b(I) Incubation of analytes, biotinylated antibodies and streptavidin conjugated magnetic particles in one step (2.5), b(II) detection via biotinylated antibody against Cy3/Cy5 and Cy3 labeled streptavidin; c(I) Incubation of analytes, biotinylated antibodies and Dy647 Streptavidin conjugated magnetic particles in one step (2.5 h).

To prevent excessive blood withdrawal from newborns the sepsis chip was miniaturized by applying a fivefold reduced sample volume. The main challenge in the realization of the miniaturized protein chip was developing the appropriate incubation and stirring system to maintain good assay performance with respect to sensitivity, reproducibility and accuracy. IC_50_ values, CVs and LODs of the resulting calibration curves were defined as criteria for assay performance. The assay designs used in the classical and miniaturized chip are presented in Figure 1. Figure 1(a) shows the classical chip format: 20 μL of patient serum sample diluted with 180 μL assay buffer are incubated and analyte concentrations are detected by incubating biotinylated detection antibodies and Dy647-labeled streptavidin. Three incubation steps are necessary to accurately run the classical chip resulting in an entire incubation time of 4 h. Figure 1(b) presents the miniaturized chip format: only 4 μL of patient serum sample diluted with 36 μL assay buffer are required to accurately run the assay with a four-fold replication. Strep.MPs are implemented in the assay for two reasons: first, to stir the small volumes during sample incubation, and secondly, to act as a detection reagent when bound to the biotinylated secondary antibodies. In the second incubation step biotinylated antibody against Cy3/Cy5 and Cy3 labeled Streptavidin are added. Hence, only two incubation steps are necessary to accurately run the miniaturized chip resulting in an entire incubation time of 3 h and 15 min.

**Figure 2.**
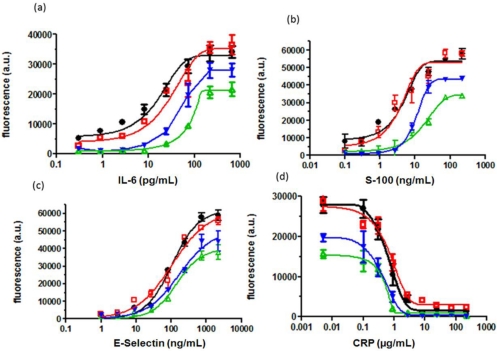
Calibration curves for (**a**) IL-6 (**b**) S-100 (**c**) E-Selectin and (**d**) CRP. The black curve ( 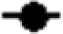) displays the 10 μL assay and the red curve ( 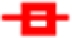) shows the 50 μL assay when adding strep.MPs; the blue curve ( 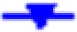) displays the classical 50 μL assay and the green curve ( 
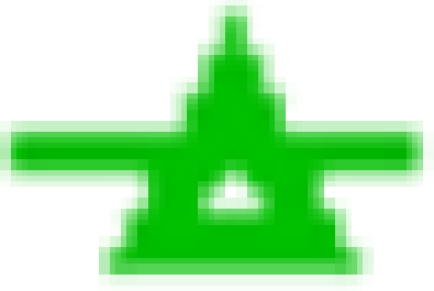
) displays the 10 μL assay without addition of strep.MPs.

Figure 2 shows the standard curves obtained for S-100, IL-6, CRP and E-Selectin when comparing the assay performance of the classical chip (50 μL) with the miniaturized chip (10 μL without strep.MPs). The IC_50_ value was taken as evaluation parameter since it lies within the ascending slope providing the highest precision of the measurement and a good reference point for comparison of different treatments. For the classical chip the IC_50_ values for IL-6, S-100, E-Selectin and CRP are 46 pg/mL, 10 ng/mL, 170 ng/mL and 4.7 μg/mL respectively, while for the miniaturized chip they were 82 pg/mL, 19 ng/mL, 202 ng/mL and 2.5 μg/mL. This demonstrates that reduction of the sample volume leads to losses in sensitivity. Obviously, sample volumes of 10 μL are too small to be properly mixed on the orbital shaker during incubation. Thus, strep.MPs were implemented as so-called stirring detection reagents in the assay to accelerate the antibody antigen kinetics. Acceleration of diffusion processes is a common issue and magnetic particles have been previously outlined as successful tool to provide sample mixing in microscale devices [21, 22]. In optical biochips, Heer *et al*. [23] reported up to a four-fold enhancement of fluorescent signals upon controlled movement of magnetic microspheres in the DNA hybridization buffer, while Smith *et al*. [18] used antibody-labeled magnetic particles to pre-concentrate the target via magnetic separation and integrate them as a tracer in the immunoassay. Oligonucleotide- [24] and antibody-labelled [25] magnetic particles were also used in magneto-resistive biochips. In our assay strept.MPs were implemented to firstly, achieve effective sample mixing and secondly, to measure antigen antibody binding events using fluorescence detection. Figure 2 shows the calibration curves obtained when adding strep.MPs to the test solution (10 μL). The assay performance was significantly enhanced in terms of the assigned IC_50_ values (16.5 pg/mL, 4.3 ng/mL, 105 ng/mL and 4.4 μg/mL for IL-6, S-100, E-Selectin and CRP).

Nano-sized and micro-sized strep.MPs (130 nm, 500 nm and 1 μm) were applied to the miniaturized assay to assess potential concerns about steric hindrance, but the assay performance was similar no matter what particle size was used.

Comparison of the miniaturized protein chip with and without strep.MPs shows that sensitivity is increased by a factor of 2 (for E-Selectin and CRP) to 4 (for IL-6 and S-100) through the addition of strep.MPs. In Section 3.2.1 this method will be further advanced in that fluorescent strep.MPs will be implemented as stirring detection reagent.

### 3.2. Optimization of the Miniaturized Chip

#### 3.2.1. Reducing Assay Steps

As outlined in Figure 1 the classical 50 μL chip (a) requires three separate incubation steps whereas the miniaturized bio-chip (b) requires only two incubation steps. In order to optimize the low volume chip the assay was performed in one step only (Figure 1(c)). This means that analytes, biotinylated antibodies and streptavidin coated magnetic particles were incubated simultaneously on the chip. Sauer *et al*. [14] showed that in the classical 50 μL format the assay performance of an “all-in-one-step” assay was significantly deteriorated compared to the three step assay. However, this does not apply to the new low volume chip, since this protocol incorporates strep.MPs which strongly promote mixing of the solution and analyte binding. Still, the detection step (Figure 1(b-II)) of the miniaturized biochip is quite complex. Therefore, magnetic particles were conjugated with dye labeled streptavidin in order to completely replace the second incubation step. Figure 2 compares the assay performance of the optimized low volume assay (black curve) with the classical assay (red curve) showing that the standard deviations, the inflection point and the maximal signals of the curves are similar. This indicates that reducing the number of assay steps has no impact on the assay sensitivity and reproducibility of results. The new low volume assay accomplishes within 2.5 h providing an accurate “all-in-one-step” assay for point-of-care diagnostics of sepsis-relevant biomarkers.

#### 3.2.2. Optimizing Incubation Time

**Figure 3.**
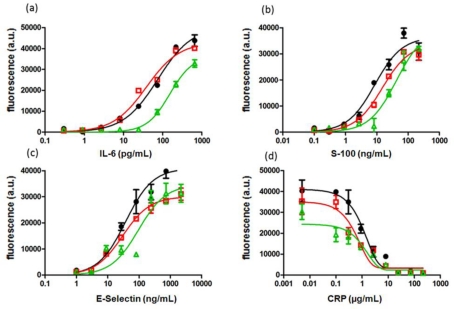
Calibration curves for (**a**) IL-6 (**b**) S-100 (**c**) E-Selectin and (**d**) CRP in human serum 1:10 diluted with assay buffer after 2.5 h ( 
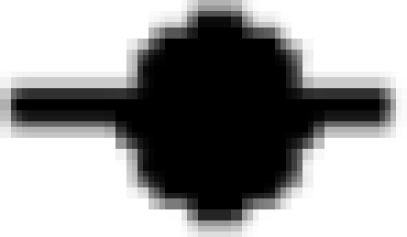
), 2 ( 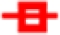) and 1.5 h ( 
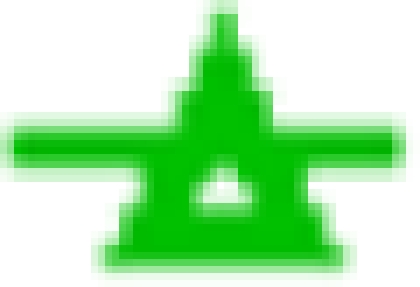
) processing time.

To guarantee a fast and effective treatment of the disease, a rapid diagnosis and thus short assay times are essential. However, in order to achieve short assay times, a compromise must be made between incubation time, assay sensitivity and data reproducibility. In order to determine the lowest incubation time necessary to perform a sensitive and accurate chip analysis three different incubation times were tested and evaluated using IC_50_, CV and LOD as measures for the assay performance. While for the detection of IL-6, S-100 and E-Selectin sandwich immunoassays were performed, a binding inhibition test was done for CRP. Figure 3 shows the calibration curves for IL-6, S-100, E-Selectin and CRP obtained with the low volume chip described in Section 3.2.1 after 1.5 h, 2 h and 2.5 h “all-in-one” incubation.

Figure 3 indicates that the effect of incubation time on the calibration curves is similar for all analyzed biomarkers. A decrease of incubation time provokes a loss in sensitivity which can be seen from the shift of calibration curve to higher analyte concentrations, reduced fluorescence signals and increased IC_50_ values. IC_50_ values are listed in Table 2 together with LODs and CVs. While sensitivity expressed by LOD, LOQ and IC_50_ is similar for IL-6 and E- Selectin after 2 h and 2.5 h incubation, there are significant differences observed for S-100 and CRP. Sensitivity was drastically reduced for all tested biomarkers, if incubated for only 1.5 h. Interestingly, reproducibility of data was not affected and remained the same (CV < 14%) regardless what incubation time or analyte was used.

In summary, a 2 h incubation step is most likely sufficient for the diagnosis of neonatal sepsis. While assay sensitivity remains unchanged for IL-6 and E-Selectin, assay sensitivity is slightly decreased for S-100 and for CRP. For diagnostic purposes the right balance between incubation time and sensitivity has to be specified individually according to the clinical requirements.

### 3.3. Quantification of “Patient” Samples

The optimized low volume protein chip as described in Section 3.2 was tested for quantification of “patient” samples and evaluated in a recovery experiment. The assay performance was assessed by recovery values calculated as the relation of interpolated and expected concentrations. Recoveries that lie within 70 and 130% are considered as acceptable in clinical diagnosis indicating reasonable accuracy of the assay [26]. Three “patient” samples with known clinically relevant concentrations of IL-6, S-100, E-Selectin and CRP were applied additionally to the standard dilution series. The concentrations of all “patient” samples were chosen to comply with real patient serum samples. Each analyte was tested in 3 different concentrations ranging from low to high clinically relevant values to cover the whole notable range.

Mean intra-array variations of 11.8%, 10.3%, 12.4% and 13.3% for IL-6, S-100, E-Selectin and CRP respectively are obtained. Average inter-array variability of the four tested markers was 11.7% which indicates a good chip-to-chip reproducibility. Figures of merit are summarized in Table 3. High accuracy (within 70 to 130%) is obtained for concentrations within the ascending part of the slope whereas recoveries obtained for especially low or high concentrations are out of the clinically acceptable range.

### 3.4. Internal Calibration Curve

**Figure 4.**
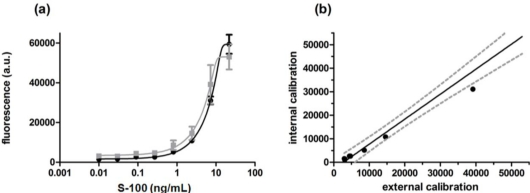
Calibration curves (**a**) and Scatter plot (**b**) for the internal ( 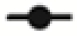) and external chip calibration ( 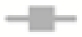). Internal and external calibration curves cover a very similar measurement range and most fluorescence intensity values cluster within the 95% confidence band (grey dotted lines) of the fit line. The coefficient of determination (R^2^) was 0.968.

For calibration of the protein microarray and quantification of the unknown sample a standard dilution series was prepared for each assay (external calibration). To do so, nine spiked antigen mixes replicated four times were applied to 36 wells. This leaves 12 wells for patient samples (e.g., three samples, four replicates) when using a set of four slides. To reduce the effort and further simplify the protein-chip the calibration was directly integrated on the chip (internal calibration). Several attempts to provide a one patient, one biochip format have been made by immobilization of the protein [27] or the detection components [28] in increasing concentrations. In our approach S-100 was conjugated to the carrier protein BSA and subsequently immobilized in increasing concentrations on ARChip Epoxy forming an internal calibration. The array of the immobilized BSA-S-100 conjugate and specific capture antibodies was then reacted with the unknown sample, biotinylated secondary antibodies and 500 nm Dy647-strep.MPs as schematically shown in Figure 2(c). Chip performance of the internal and external calibration curve was compared. As can be seen from Figure 4, both internal and external calibration curves are very similar in terms of shape, measurement range and sensitivity as defined by the slope of the curve which demonstrates the suitability of the approach. Nevertheless an internal standard curve is advantageous over an external curve as it needs less reagents and shorter handling time and allows faster availability of test results: while quantification via an external calibration curve requires nine wells per slide and one well for the sample of interest, integration of an internal standard curve reduces the number of wells per slide to only one well, the sample well.

**Figure 5.**
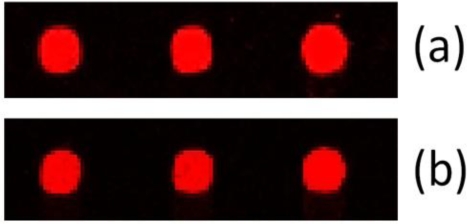
Processed chip showing spots obtained for the external (**a**) and internal (**b**) calibration at a concentration of 8.1 ng/mL S-100.

Figure 4(a) compares the curves obtained for the internal and external calibration, and Figure 5 shows a typical fluorescence image of processed calibration spots. The image demonstrates the high quality of the arrayed spots which is depicted by the regular spot morphology, the concise shape and the even distribution within the spot. This is also reflected in the good reproducibility of biomarker measurement as can be seen from Table 3: the coefficient of variation is ≤14%.

The calibration curves are almost identical both in terms of shape and maximal signal intensities which is in accordance with the assay parameters obtained (internal calibration: LOD 0.239 ng/mL, LOQ 1.152 ng/mL, IC_50_ 3.38 ng/mL, CV_inter_ 12%; external calibration: LOD 0.241 ng/mL, LOQ 0.976 ng/mL, IC_50_ 2.84 ng/mL, CV_inter_ 13%). Figure 4(b) depicts the correlation and precision between internal and external calibration. The fluorescence signals obtained for the external calibration are plotted against the fluorescence signals from the internal calibration. A strong linear correlation (coefficient of determination (R^2^) of 0.968) was observed: most values cluster within the 95% confidence band (grey dotted lines) of the fit line. The significance of the linear correlation is confirmed by a p-value below 0.01%. The mean intra-array variation was 10.4% for the external and 10.8% for the internal calibration.

Additionally to the standard dilution series “patient” samples with known analyte concentrations (2.5 ng/mL and 12.5 ng/mL) were processed on the chip to evaluate the accuracy of the calibration curves calculated from the %-relation between interpolated concentrations and expected concentrations. The recoveries obtained were 70% and 103% when interpolating to the external calibration and 70% and 83% when interpolating to the internal calibration. The recoveries are within 70–130% which is appropriate for clinical purposes.

## 4. Conclusions

We have presented herein a protein chip that fulfills the requirements for point-of-care diagnostics in terms of miniaturization, assay time and internal calibration. A sample volume of 40 μL which corresponds to 4 μL patient serum sample diluted 1:10 with assay buffer is sufficient to quantify sepsis associated serum proteins. The optimized low volume chip is especially attractive for diagnosis in neonates since the reduced sample volume minimizes stress and pain caused through excessive blood withdrawal. Sample mixing to overcome diffusion limitations is ensured through the addition of streptavidin coated magnetic particles (strep.MPs) acting as both stirring and detection components. Fluorescence labeling of the streptavidin coated magnetic particles further accelerates and simplifies the assay assembly resulting in a single step assay that is accomplished within 2.5 h. We demonstrated that for some analytes (exemplarily shown for IL-6 and E-Selectin) an incubation time of 2 h is sufficient to allow sensitive and accurate quantification. For some analytes (exemplarily S-100 and CRP) shortening of the incubation time to 2 h accounts for decreased sensitivity calling for variation of the incubation time according to the requirements with respect to assay sensitivity and assay time. The establishment of an internal calibration makes the protein chip even more attractive for point-of-care applications since it further simplifies the device in terms of quality control and handling. The low volume protein-chip allows accurate determination of the sepsis biomarkers with good reproducibility (CV < 14%). Recoveries are within the clinically acceptable range (70–130%).

## Figures and Tables

**Table 1.** Cut-off values for the respective biomarkers.

**Biomarker**	**Cut-off values**	**Biomarker**	**Cut-off values**
IL-6 (pg/mL)	18 [7], 31 [6]	PCT (pg/mL)	500 [9], 36 [13]
IL-8 (pg/mL)	20 [12]	Neopterin (pg/mL)	16 [11]
IL-10 (pg/mL)	17 [13]	S-100 (ng/mL)	10.4 [10]
TNF alpha (pg/mL)	17 [6]	CRP (μg/mL)	10 [7,9] 12 [6]
E-Selectin (ng/mL)	174 [6]		

**Table 2.** Assay parameters for IL-6, S-100, E-Selectin and CRP obtained after 2.5 h, 2 h and 1.5 h incubation.

**Analyte**	**IL-6 (pg/mL)**			**S-100 (ng/mL)**			**E-Selectin (ng/mL)**			**CRP (μg/mL)**		
Time	2.5 h	2 h	1.5 h	2.5 h	2 h	1.5 h	2.5 h	2 h	1.5 h	2.5 h	2 h	1.5 h
LOD	1.1	1.2	149.0	0.7	1.8	5.0	4.0	6.3	30.1	0.10	0.27	0.48
LOQ	6.4	7.0	151.5	1.6	7.1	14.7	12.6	11.4	41.7	0.22	0.42	0.87
IC_50_	68	70	152	9	15	43	31	30	96	30	64	138
CV (%)	11	10	14	10	8	14	11	12	11	10	11	13

**Table 3.** Recoveries, LODs, LOQs and inter- and intra-array variabilitiy for the optimized low volume assay.

**Biomarker**	**Conc_exp_**	**Conc_calc_**	**Recovery (%)**	**LOD**	**LOQ**	**CV_inter_ (%)**	**CV_intra_ (%)**
IL-6 (pg/mL)	1.50	1.2	79	1.2	6.5	10.7	11.8
	7.5	6.6	88				
	37.5	64.5	172				

S-100 (ng/mL)	0.50	- (*)	-	0.89	5.3	10.0	10.3
	2.5	1.7	69				
	12.5	11.7	94				

E-Selectin (ng/mL)	4.50	4.2	92	2.7	4.5	12.2	12.4
	22.5	16.8	75				
	112.5	103.0	92				

CRP (μg/mL)	0.5	1.0	200	0.13	0.32	14.0	13.3
	2.5	2.6	102				
	12.5	- (*)	-				

(*) out of the range of the calibration curve.
